# Evidence for L1-associated DNA rearrangements and negligible L1 retrotransposition in glioblastoma multiforme

**DOI:** 10.1186/s13100-016-0076-6

**Published:** 2016-11-11

**Authors:** Patricia E. Carreira, Adam D. Ewing, Guibo Li, Stephanie N. Schauer, Kyle R. Upton, Allister C. Fagg, Santiago Morell, Michaela Kindlova, Patricia Gerdes, Sandra R. Richardson, Bo Li, Daniel J. Gerhardt, Jun Wang, Paul M. Brennan, Geoffrey J. Faulkner

**Affiliations:** 1 Mater Research Institute - University of Queensland, TRI Building, Woolloongabba, QLD 4102 Australia; 2 BGI-Shenzhen, Shenzhen, 518083 China; 3 Department of Biology and the Novo Nordisk Foundation Center for Basic Metabolic Research, University of Copenhagen, Copenhagen, 1599 Denmark; 4 School of Chemistry and Molecular Biosciences, University of Queensland, Brisbane, QLD 4072 Australia; 5 Edinburgh Cancer Research Centre, IGMM, University of Edinburgh, Edinburgh, EH42XR UK; 6 Queensland Brain Institute, University of Queensland, Brisbane, QLD 4072 Australia

## Abstract

**Background:**

LINE-1 (L1) retrotransposons are a notable endogenous source of mutagenesis in mammals. Notably, cancer cells can support unusual L1 retrotransposition and L1-associated sequence rearrangement mechanisms following DNA damage. Recent reports suggest that L1 is mobile in epithelial tumours and neural cells but, paradoxically, not in brain cancers.

**Results:**

Here, using retrotransposon capture sequencing (RC-seq), we surveyed L1 mutations in 14 tumours classified as glioblastoma multiforme (GBM) or as a lower grade glioma. In four GBM tumours, we characterised one probable endonuclease-independent L1 insertion, two L1-associated rearrangements and one likely *Alu*-*Alu* recombination event adjacent to an L1. These mutations included PCR validated intronic events in MeCP2 and EGFR. Despite sequencing L1 integration sites at up to 250× depth by RC-seq, we found no tumour-specific, endonuclease-dependent L1 insertions. Whole genome sequencing analysis of the tumours carrying the MeCP2 and EGFR L1 mutations also revealed no endonuclease-dependent L1 insertions. In a complementary in vitro assay, wild-type and endonuclease mutant L1 reporter constructs each mobilised very inefficiently in four cultured GBM cell lines.

**Conclusions:**

These experiments altogether highlight the consistent absence of canonical L1 retrotransposition in GBM tumours and cultured cell lines, as well as atypical L1-associated sequence rearrangements following DNA damage in vivo.

**Electronic supplementary material:**

The online version of this article (doi:10.1186/s13100-016-0076-6) contains supplementary material, which is available to authorized users.

## Background

Glioblastoma multiforme (GBM) is the most common and aggressive brain tumour in adults [1]. Ninety-five percent of diagnosed GBM tumours originate *de novo* (primary GBM), while the remainder progress from a lower grade glioma (secondary GBM) [2]. Primary and secondary GBM tumours are histologically indistinguishable [3]. To date, genomic analyses have elucidated somatic mutations and intra-tumoural heterogeneity governing GBM progression and resistance to therapy [4–6]. Defects in several DNA repair mechanisms, especially in the repair of DNA double strand breaks (DSBs), are known to enable genomic aberrations, such as deletions and amplifications, in GBM [7, 8]. Despite the extensive genomic analyses performed thus far, the GBM genome may yet harbour additional etiological clues that could improve treatment and patient outcomes.

L1 retrotransposons are endogenous mutagens known to cause sporadic disease, including cancer [9]. A full-length human L1 is ~6 kb long [10, 11] and contains a 5′ untranslated region (UTR), two non-overlapping open reading frames that encode respectively for a 40KDa RNA binding protein (ORF1p) [12, 13] and a 150KDa protein with both endonuclease (EN) and reverse transcriptase (RT) activities (ORF2p) [14, 15], and a 3′UTR. The L1 5′UTR bears an internal promoter with sense and antisense activity [16, 17] and a recently described antisense open reading frame (ORF0) [18]. Canonical L1 mobilisation depends on the transcription and translation of L1 and the formation of a ribonucleoprotein particle (RNP) consisting of ORF1p and ORF2p, and their encoding mRNA. Once the RNP enters the nucleus, the L1-encoded EN can cleave genomic DNA [15] and, typically, generate a new L1 insertion via target-primed reverse transcription (TPRT) [19]. Hallmarks of L1 integration by TPRT include use of an L1 EN recognition motif (5′-TT/AAAA), target site duplications (TSDs), and an L1 poly-A tail [20]. Endonuclease-independent (ENi) L1 mobilisation can also occur into pre-existing DNA double strand breaks, producing insertions that lack TPRT hallmarks [21–24]. Notably, L1 can mobilise other polyadenylated RNAs, such as *Alu* retrotransposons, in *trans* [25–27]. L1 and *Alu* elements can also participate in DNA rearrangements driven by recombination [28, 29]. Although TPRT-mediated L1 mobilisation occurs in many cancers [30–38] and neural cells [39–42], several recent studies employing high-throughput sequencing have reported a surprising absence of somatic L1 insertions in brain tumours [6, 30–32, 35].

We hypothesised that L1-associated DNA rearrangements in GBM might occur via recombination or an atypical retrotransposition mechanism and therefore may lack the TPRT hallmarks required for L1 insertion recognition by previous genomic analyses. Alternatively, we considered that L1 insertions in GBM could be restricted to sub-clonal and highly heterogeneous events. We therefore applied deep retrotransposon capture sequencing (RC-seq) [34, 42] to 14 brain tumour patients (9 GBM and 5 lower grade glioma) and detected tumour-specific L1-associated mutations lacking TPRT hallmarks in 4 GBM tumour samples, and also found no examples of TPRT-driven L1 mobilisation. Complementary assays using an engineered L1 reporter assay [43] revealed negligible in vitro L1 activity in all tested GBM cell lines. These experiments confirm that L1 mobilisation is absent or very rare in GBM tumours and cell lines. Unusual endonuclease-independent L1 retrotransposition or L1-associated recombination events can however occasionally occur, and may impact the expression of genes relevant to GBM aetiology and neural cell morphology.

## Methods

### Patient samples

Tissues were obtained from 14 patients undergoing surgical removal of a brain tumour at the Department of Clinical Neurosciences, Western General Hospital, Edinburgh, UK. All patients gave informed consent for tumour and peri-tumoural tissue removed in the normal course of surgery, and blood obtained intra-operatively, to be used for research. Ethical approval for the study was granted to P.M.B. by the East of Scotland Research Ethics Service (SR018). Tissue designation as ‘tumour’ or ‘adjacent brain’ was determined at the time of sampling based on pre-operative imaging, intra-operative image guidance and macroscopic inspection. Blood was sampled from 9 patients (Additional file 1: Table S1) intra-operatively and stored in lithium/heparin tubes. Tissue and blood samples were snap frozen on dry ice within 30 min of sampling and stored at −80 °C. Ethical approval for subsequent experiments performed at the Mater Research Institute – University of Queensland was granted to G.J.F. by the Mater Health Services Human Research Ethics Committee (Reference: 1915A) and the University of Queensland Human Research Ethics Committee (Reference: 2014000221). From all samples, genomic DNA was isolated by standard phenol-chloroform extraction.

### RC-seq libraries and sequencing

Paired-end 150mer multiplexed Illumina libraries were constructed from genomic DNA samples as described previously [34], with the following minor modifications: sonicated DNA was size selected for fragments of 230–260 bp by gel purification and used as template for 10 cycles of ligation-mediated PCR (LM-PCR). Libraries were quantified and insert size confirmed using an Agilent Bioanalyzer 2100 with a DNA1000 chip (Agilent Technologies, USA). L1 enrichment was achieved via two different RC-seq capture designs. Equimolar quantities of tumour and adjacent brain libraries from patients #1-#5 were pooled and hybridised to a second generation (V2) RC-seq capture pool [34] composed of 80 biotinylated oligonucleotide probes tiled across the L1-Ta consensus sequence L1.4 [44] 5′ and 3′ ends (Additional file 2: Figure S1A). Hybridisation and library processing were performed as described previously [34]. L1 enriched libraries were sequenced on Illumina HiSeq2000 platform (BGI-Shenzhen, China).

**Fig. 1:**
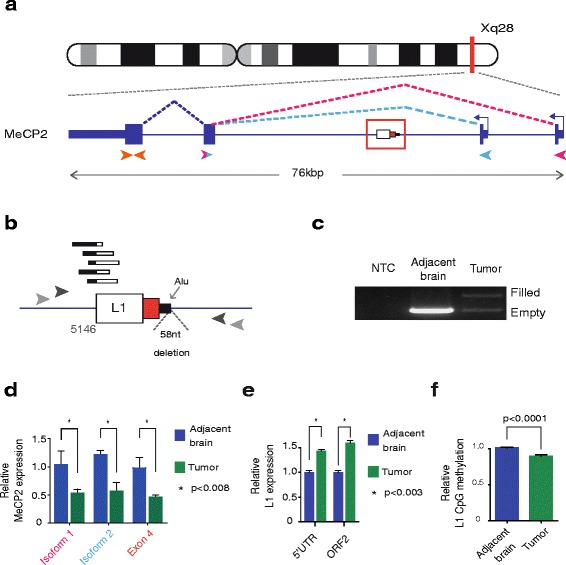
Characterisation of a somatic L1-associated DNA rearrangement within MeCP2. **a** Patient #2 MeCP2 mutant allele: a 0.9 kb L1PA2 sequence antisense to MeCP2. Direction of transcription (*blue arrows*), transcript isoforms (*purple/pink lines*) and qRT-PCR primers for MeCP2 expression assays (*arrowheads*) are indicated. **b** L1 mutation magnified view: RC-seq reads detected at the L1 5′ terminus (*black/white bars*). The L1 sequence comprises a truncated fragment of L1 ORF2 (*white box*), the 3′UTR without a poly-A tail (*red box*) and 37 nt from an Alu (*black box*). A 58 nucleotide deletion was also identified (triangle). Primers used for PCR validation are indicated as grey arrows. **c** Mutation site PCR validation: the mutant MeCP2 allele carrying L1 (filled) was only detected in patient #2 tumour whilst the empty site was found in both tumour and adjacent brain samples. No amplification was detected when water was used as template (NTC). **d** qRT-PCR measurement of MeCP2 transcript isoforms: The relative levels of RNA from both isoforms were significantly reduced in tumour (*blue*) versus adjacent brain (*green*) samples. Data for each group were normalised to non-tumour values, pooled and presented as mean +/− SEM (*p < 0.008, two tailed *t*-test, df = 6). Text colour relates with the primer pair used as represented in (a). **e** qRT-PCR measurement of L1 transcript abundance measured at the L1 5′UTR and ORF2 regions: The relative levels of RNA from both regions were significantly increased in tumour (*blue*) versus adjacent brain (*green*) samples. Data for each group were normalised to adjacent brain values, pooled and presented as mean +/− SEM (*p < 0.001, two tailed *t*-test, df = 10). **f** L1 promoter methylation: CpG methylation was measured across the L1 promoter CpG-island sequence. Tumour samples (*blue*) showed reduced methylation when compared to adjacent brain samples (*green*). Data for each group were normalised to non-tumour values, pooled and presented as mean +/− SEM (*p < 0.001, paired *t*-test, df = 18)

Three additional library pools comprising i) tumour and adjacent brain from patients #1-#7, ii) tumour and adjacent brain from patients #8-#14 and iii) blood from patients #6-#12 and #14 were hybridised using a third generation (V3) RC-seq capture protocol involving only two optimised, custom locked nucleic acid (LNA) oligonucleotide probes (Exiqon Vedbaek, Denmark) respectively targeting the 5′ and 3′ ends of L1.4 [42] (Fig. 1a). LNA probe LNA-D/5Biosg/CTCCGGT + C + T + ACAGCTC + C + C + AGC targeted the 5′ end and LNA-B/5Biosg/AG + A + TGAC + A + C + ATTAGTGGGTGC + A + GCG targeted the 3′ end (+ denotes LNA positions within each probe). Pools i) and ii) were sequenced on an Illumina HiSeq2500 (Ambry Genetics, USA). Pool iii) was sequenced by multiple Illumina MiSeq runs. A total of 3,252,752,806 2x150mer RC-seq reads were generated (Additional file 1: Table S1). RC-seq FASTQ files are available from the European Nucleotide Archive (ENA) under the identifier PRJEB1785.

### RC-seq bioinformatic analysis

RC-seq read data were analysed using TEBreak (https://github.com/adamewing/tebreak/tree/f7f01c1) with settings –mincluster 2, −minclip 30, −minq 1. Briefly, TEBreak aligned RC-seq reads against the human reference genome (hg19) using BWA-MEM [45] with settings –Y and –M to output soft-clipped secondary alignments. PCR duplicates were marked with MarkDuplicates from the Picard Tools library (http://broadinstitute.github.io/picard/). Non-duplicate reads that aligned partially to the reference genome but had ≥30 nt soft-clipped from either end were retained; clipped ends were then aligned against L1.4 using the same BWA-MEM settings. Split-read mappings joining an L1 to the reference genome were then clustered and annotated for the presence of TSDs or deletions. Clusters with at least two reads supporting a consistent breakpoint were output in VCF format and further post-processed using the summary.py script included in the TEBreak distribution. This further filtered candidates by ensuring a consistent breakpoint between BWA-MEM and BLAT [46] alignments, excluded clusters mapping to locations in the reference genome occupied by other L1s and required that the consensus sequence of each cluster matched L1.4 by at least 90 % over ≥30 nt and the reference genome by at least 95 % based on the BLAT alignment.

RC-seq sensitivity for each library was assessed based on a cohort [41] of 960 reference genome L1-Ta and L1 pre-Ta insertions with intact 3′ ends (Evrony et al. Table S5, “Category 4”) detected by ≥20 and ≥8 reads for patients #1-#5 and #6-#14, respectively (Additional file 1: Table S1). Sensitivity was further assessed in terms of polymorphic L1 insertions detected in each individual, with ≥10 and ≥4 reads required in both the tumour and other (adjacent brain and blood) libraries for patients #1-#5 and #6-#14, respectively, to report an L1 insertion (Additional file 1: Table S1). These thresholds exceed those used in another recent work, where we reported a 98.5 % validation rate for polymorphic L1 insertions detected by 2 RC-seq reads and tested by PCR [34]. RC-seq coverage statistics presented in Additional file 1: Table S1 were calculated as the total number of RC-seq reads in a given library that spanned a 5′ or 3′ L1-genome junction of the abovementioned cohort of 960 reference genome L1 insertions, divided by 960 to generate an average value. Non-reference L1 insertions were annotated as tumour-specific if they were: i) found in only one tumour sample with ≥8 RC-seq reads and >10× more RC-seq reads than all other samples combined, ii) absent from published L1 polymorphism databases [30, 32–35, 47–51], iii) likely to be found by the corresponding RC-seq capture design (a 5′ L1-genome junction for an L1 > 6000 nt, <1000 nt in length or a 3′ L1-genome junction), iv) with TEBreak ‘strand confidence’, ‘family confidence’ and ‘position confidence’ scores of >0.9, >0.9 and >0.3, respectively, and v) presented microhomology of <10 nt between the integration site and L1.4 (to exclude possible molecular chimeras). Four putative tumour-specific L1 mutations were reported at these thresholds (Additional file 1: Table S2).

### L1 mutation PCR validation

Empty/filled site PCR assays were used to validate tumour-specific L1 mutations detected by RC-seq. Primers flanking either side of each insertion were designed using Primer3 [52] (Additional file 1: Table S3). PCR reactions involved the following reagents: 1U MyTaq DNA polymerase (Bioline, Australia, #BIO-21106), 1× MyTaq Reaction Buffer, 2 μM primers and 20 ng template DNA in a 25 μL reaction volume with the following cycling conditions for the MeCP2 L1 mutation: 3 min at 92 °C, then 10 cycles of 30s at 92 °C, 30s at 60 °C and 6 min 30s at 68 °C, followed by 20 cycles of 30s at 92 °C, 30s at 58 °C and 6 min 30s at 68 °C (increasing by 20s per cycle), followed by a single extension step at 68 °C for 10 min. Products were treated with ExoSap-IT (Affymetrix, USA), with 2 μL of product then used for a second PCR reaction with the same conditions as the first round, except with 30 cycles in the second phase. For the EGFR L1 mutation, PCR was performed using primers targeting the 5′ L1-genome junction with the following cycling conditions: 2 min at 95 °C, then 20 cycles of 15 s at 95 °C, 30s at 59 °C and 30s at 72 °C, followed by a single extension step at 72 °C for 10 min. Products were again treated with ExoSap-IT, with 2 μL of product used for a second PCR reaction with the same conditions as the first round, except with 30 cycles. PCR products were capillary sequenced using an ABI3730 (AGRF, Brisbane, AUS) and the results are provided in Additional file 1: Table S3.

### qRT-PCR analyses

Snap frozen tumour and adjacent brain tissues from patient #2 were shaved with a scalpel on dry ice and re-suspended in Trizol Reagent (Invitrogen, Life Technologies, USA, #15596-026) following manufacturer’s instructions for total RNA isolation. Quantification was performed using NanoDrop 1000 (Thermo Fisher Scientific, USA). 2 μg total RNA was treated with DNase I (Ambion, Life Technologies, USA, #AM1906) and used as template for cDNA synthesis with SuperScript III Reverse Transcriptase (Invitrogen, Life Technologies, USA, #18080-093) following manufacturer’s instructions. 2 μg total RNA was processed as described with no reverse transcriptase added to the cDNA synthesis reaction for further use as negative control (RT-). cDNA from adjacent brain tissue was diluted to final concentrations of 1:5, 1:10, 1:20, 1:40 and 1:80 and used to generate a standard curve for each primer set. Samples were diluted to 1:20 final concentration for qRT-PCR. Real time PCRs were performed using SensiFast SYBR Lo-ROX kit (Bioline, Australia, #BIO-94005) and run on a ViiA 7 Real-Time PCR System (Life Technologies, USA) with standard curve experiment analysis settings. Negative control qRT-PCRs were performed using water as template (no template control, NTC) and 2 μl of RT- reaction; no amplification was detected. MeCP2 isoforms (NM_004992) were assessed using 5′-GAGGCGAGGAGGAGAGAC and 5′-TGGTAGCTGGGATGTTAGGG as forward primers for isoforms 1 and 2, respectively, and 5′-GCAGAGTGGTGGGCTGAT as a common reverse primer to amplify 154 nt of isoform 1 and 161 nt of isoform 2. Additionally, 143 nt of the MeCP2 exon 4, present in both isoforms, were amplified using 5′-CAGAGGAGGCTCACTGGAGA as forward primer and 5′-GGCATGGAGGATGAAACAAT as reverse primer. EGFR (NM_005228) was amplified on the 5′UTR (amplicon of 156 nt) and the junction between exons 11 and 12 (176 nt) with the following primers:

- EGFR 5′UTR, 5′-CCAGTATTGATCGGGAGAGCC, 5′-CTCGTGCCTTGGCAAACTTTC
- EGFR exon 11–12 junction, 5′-GACCAAGCAACATGGTCAGT, 5′-TTTTCTGACCGGAGGTCCCA

L1 (L1.4) expression was assessed by targeting 61 nt of the 5′UTR and 85 nt of the ORF2 with the primers: L1 5′UTR:

- 5′-ACAGCTTTGAAGAGAGCAGTGGTT, 5′-AGTCTGCCCGTTCTCAGATCT
- L1 ORF2: 5′-TGCGGAGAAATAGGAACACTTTT, 5′-TGAGGAATCGCCACACTGACT

156nt of TATA-binding protein mRNA (TBP, NM_003194) and 173 nt of beta actin mRNA (ACTB, NM_001101) were amplified with the following primers:

- TBP, 5′-GCAAGGGTTTCTGGTTTGCC, 5′-GGGTCAGTCCAGTGCCATAA
- ACTB, 5′-AGAAAATCTGGCACCACACC, 5′-TAGCACAGCCTGGATAGCAA

Standard curve parameters for qRT-PCR (slope, y-intercept, r^2^) are as follows: MeCP2 isoform 1: −3.312, 29.329, 0.95; MeCP2 isoform 2: −2.946, 29.578, 0.943; MeCP2 exon 4: −3.796, 27.991, 0.897; EGFR 5′UTR: −4.057, 30.028, 0.931; EGFR exon 11–12 junction: −4.660, 28.317, 0.985; TBP: −3.754, 27.208, 0.973; L1 5′UTR: −3.094, 21.209, 0.996; L1 ORF2: −3.11, 18.836, 0.9992; ACTB: −3.954, 22.606, 0.999.

Relative expression levels were calculated using five technical replicates and normalised to TBP (for MeCP2 and EGFR) or ACTB (for L1). Statistical analysis was performed with Prism5 (GraphPad Software), applying a *t*-test with a 99 % confidence interval.

cDNA from patient #2 tumour was used as template for RT-PCR to amplify possible L1-MeCP2 chimeric transcripts. The following chimeric variants were tested; Exon 1–L1, using a forward primer within exon 1 5′-GAGGCGAGGAGGAGAGAC and a reverse primer within the new L1, 5′-CACCAGCATGGCACATGTAT. Exon 2–L1, forward primer (5′-TGGTAGCTGGGATGTTAGGG) in exon 2 and reverse primer within L1 (5′-CACCAGCATGGCACATGTAT). L1-Exon 3, with the L1 primer as a reverse primer (5′-GCACATTGTGCAGGTTAGTTAC) and a forward primer within exon 3, (5′-GCAGAGTGGTGGGCTGAT).

### MeCP2 deletion quantification

5 ng of genomic DNA from patient #2 adjacent brain and tumour were used as a template for a PCR to amplify the deleted region within MeCp2 using 5′-AAATTAGCCAGGCGTGGTG as forward primer within the deleted region and 5′-TCCTGTTTTGTCTTACGTCTTGA as reverse primer downstream of the deleted region. The PCR conditions were as follow; 1U MyTaq DNA polymerase (Bioline, Australia, #BIO-21106), 1× MyTaq Reaction Buffer, 2 μM primers and 5 ng template DNA in a 25 μL reaction volume with the following cycling conditions: 3 min at 92 °C, then 35 cycles of 15 s at 92 °C, 15 s at 56 °C and 15 s at 72 °C, followed by a single extension step at 72 °C for 10 min. PCR amplicons were resolved in a 2 % agarose gel and scanned (Typhoon FLA 9500, GE Healthcare life science, US). Amplicons present on the scanned picture was quantified using Image Studio Lite version 4 software (LI-COR Biosciences). Values were corrected for background and normalised to adjacent brain value.

### L1 promoter methylation

200 ng of genomic DNA extracted from tumour and adjacent brain tissues was bisulfite converted using EZ DNA Methylation-Lightning Kit (Zymo Research, CA, USA) following manufacturer’s instructions. After purification, 2 μL was used as template for a PCR reaction using L1-Bis-F and L1-Bis-R primers as described by Shukla et al. [34]. PCR reactions were performed using MyTaq DNA polymerase (Bioline, Australia, #BIO-21106) in a 25 μL volume with the following cycling conditions: 2 min at 95 °C, then 25 cycles of 15 s at 95 °C, 60s at 55 °C and 60s at 72 °C, followed by a single extension step at 72 °C for 10 min. The ~350 bp PCR product was gel purified using QIAquick gel extraction kit (QIAGEN, NLD). Illumina libraries were generated for each purified PCR product using the NEBNext Ultra DNA Library Prep Kit (New England Biolabs Inc., USA) following manufacturer’s instructions and sequenced on an Illumina MiSeq. 250mer paired-end reads were assembled into single contigs using FLASH [53] (−m 15 -M 150 -× 0.3). Contigs were then aligned to the mock bisulfite converted L1-Ta consensus L1.4 using blastn (−dust no -penalty −1 -gapopen 2 -gapextend 1 -max_target_seqs 1). The methylation status of CpG sites in the L1.4 promoter CpG island was used to compare tumour and adjacent brain L1 promoter methylation, as performed previously [34]. Mutated CpG dinucleotides were excluded from analysis, as were reads with less than 95 % conversion of non-CpG cytosines.

### Patient #2 and #8 whole genome sequencing and analysis

Illumina libraries (TruSeq Nano DNA sample preparation kit) were generated from patient #2 tumour and adjacent brain and, for patient #8, tumour and blood genomic DNA. Libraries had an insert size of ~300 nt and were sequenced on an Illumina HiSeq X Ten platform (Kinghorn Centre for Clinical Genomics, Garvan Institute of Medical Research, Australia). Reads were aligned to hg19 using BWA-MEM (parameters –Y –M –R < read group name>) and sorted using SAMtools [45]. PCR duplicates were marked using MarkDuplicates and local INDEL realignment was carried out with GATK 3.3. Patient #2 adjacent brain and tumour libraries were sequenced to 44.3× and 84.6×, respectively. Patient #8 blood and tumour libraries were sequenced to 59.8× and 124.2× aligned read depth, respectively. Point mutations and short insertions/deletions were detected using Strelka [54] and Platypus [55]. Structural rearrangements were detected using Delly [56] and Manta [57] and somatic CNVs were detected using cn.MOPS [58], cross-referenced with SV calls, and manually inspected (Additional file 3: Figure S3, Additional file 4: Figure S4, Additional file 5: Figure S5 and Additional file 6: Figure S6). WGS FASTQ files are also available from the ENA under the identifier PRJEB1785.

### Cell culture

GBM cell lines were purchased from American Type Culture Collection (ATCC, USA) and grown in a humidified, 5 % CO2 incubator at 37 °C in the complete media as described by the provider. DBTRG-05MG (ATCC, USA, #CRL-2020) cells were grown in ATCC-formulated RMPI-1640 medium (Gibco, Life Technologies, USA, #A10491-01) supplemented with 10 % foetal bovine calf serum (Gibco, Life Technologies, USA, #16000044), non-essential amino acids (Gibco, Life Technologies, USA, #11140050) and 100 U/mL penicillin, 0.1 mg/mL streptomycin (Gibco, Life Technologies, USA, #15140122) to generate complete media. M059J (ATCC, USA, #CRL-2366) cells were grown in media containing a 1:1 mixture of Dulbecco’s Modified Eagle’s Medium and Ham’s F12 Medium (DMEM-F12, ATCC, USA, #30-2006) supplemented with 10 % foetal bovine calf serum, non-essential amino acids and 100 U/mL penicillin, 0.1 mg/mL streptomycin. LN18 (ATCC, USA, #CRL-2610) and LN229 (ATCC, USA, #CRL-2611) were grown in Dulbecco’s Modified Eagle’s Medium (DMEM, ATCC, USA, #30-2002) supplemented with 5 % foetal bovine calf serum and 100 U/mL penicillin, 0.1 mg/mL streptomycin. HeLa cells were grown in a humidified, 5 % CO2 incubator at 37 °C in the complete media, Dulbecco’s Modified Eagle’s Medium (DMEM, ATCC, USA, #30-2002) supplemented with 10 % foetal bovine calf serum and 100 U/mL penicillin, 0.1 mg/mL streptomycin.

### Generation of L1 retrotransposition assay plasmids

Plasmids carrying i) an L1 EN mutant (pCEP4-L1.3D205A) [23] and ii) an L1 reverse transcriptase mutant (JJ105-L1.3-D702A) [59, 60] were digested with *NotI-Hf* and *BstZ17I* restriction enzymes (New England Biolabs, USA, #R3189 and #R0594) at 37 °C for 2 h to obtain L1.3-D205A and the JJ-*NotI-Hf/BstZ17I* backbone. This backbone was also treated with alkaline phosphatase (New England Biolabs, USA, #M0290) for 30 min at 37 °C to use for subsequent cloning. Purified L1.3-D205A was ligated to the JJ-*NotI-Hf/BstZ17I* backbone using T4 ligase (New England Biolabs, USA, #M0202) for 2 h at room temperature generating JJ-L1.3-D205A. To obtain wild-type L1.3 (L1.3 WT) [61] and L1.3-D205A/D702A (EN and RT double mutant) constructs, plasmids carrying the individual mutations were digested with *EcoRI* (New England Biolabs USA, #R3101), *NotI* and *BstZ17I* simultaneously (2 h at 37 °C). Fragments corresponding to L1.3-*NotI*/*EcoRI*, L1.3-D205A-*NotI*/*EcoRI*, L1.3-*EcoRI*/*BstZ17I* and L1.3-D702A-*EcoRI*/*BstZ17I* were gel purified. L1.3-*NotI*/*EcoRI* and L1.3-*EcoRI*/*BstZ17I* were ligated to JJ-*NotI-Hf/BstZ17I* backbone to obtain JJ-L1.3 plasmid. JJ-L1.3-D205A-D702A was generated by ligation of L1.3-D205A-*NotI*/*EcoRI* and L1.3-D702A-*EcoRI*/*BstZ17I* to the JJ-*NotI-Hf/BstZ17I* backbone. Ligations were transformed in One Shot TOP10 Chemically Competent *E. coli* bacteria (Invitrogen, Life Technologies, USA, #C4040), and plated in LB-agar (Sigma-Aldrich, USA, #L2897) and 100 μg/ml ampicillin (Sigma-Aldrich, USA, #A9518). All plasmid sequences were confirmed by capillary sequencing.

### L1 retrotransposition assay

Plasmid DNA was purified on maxiprep columns (Qiagen, NED, #12143) and diluted in sterile dH_2_O to 0.5 μg/μL. GBM cells were seeded in 6-well dishes in their respective complete media to ~25 % confluence. Cells were transfected at the time of seeding with FuGENE HD transfection reagent (Promega, USA, #E2312) following the manufacturer’s protocol using 1:4 DNA: FuGENE ratio. Each transfection well received 1 μg plasmid DNA, 4 μL FuGENE reagent and 2 mL of complete media. Media was changed 24 h post transfection and selection with blasticidin S HCl (Life technologies, USA, #A11139) began 4 days post transfection. Cells were selected for antibiotic resistance for 10 days using a final concentration of 5 μg/ml for HeLa, DBTRG-05MG and LN229 and 2 μg/mL for LN18 and M059J.13-14 days post transfection, cells were washed twice with 1xPBS and fixed and stained as described by Moran et al. [43].

Transfection efficiency for each plasmid was calculated by flow cytometry (BD FACSCanto II, BD Bioscience, USA). GBM cell lines were co-transfected as described above with 0.5 μg of each JJ construct and 0.5 μg of pCAG-GFP (plasmid that constitutively expresses GFP). 72 h post-transfection, cells were harvested and re-suspended in 1× PBS. Propidium iodide (Thermo Fisher, Life Technologies, USA, #P3566) was added to the samples for identification of dead cells. GFP positive cells were gated based on fluorescence of untransfected cells and transfection efficiency calculated as the percentage of GFP positive cells using FlowJo 10.0.8 software (FlowJo LLc., USA) (Additional file 7: Figure S2, Additional file 1: Table S4).

## Results

### L1 mutations identified in 4 GBM tumours

We applied RC-seq to 9 GBM and 5 lower grade glioma sample sets, including tumour and matched adjacent brain or blood (Additional file 2: Figure S1A, Additional file 1: Table S1). Tumour and non-tumour samples from five of these patients were sequenced to ~250× coverage of targeted L1-genome junctions by RC-seq, while samples from the remaining individuals were sequenced to ~55× coverage. Overall, we detected 93.6 % of 960 reference genome copies [41] of the most active human L1 subfamily, L1-Ta, as well as an average of 208 polymorphic L1-Ta insertions per sample (Additional file 1: Table S1), despite stringent RC-seq reporting thresholds (see Materials and Methods).

We identified four putative tumour-specific L1 mutations in four different GBM patients (Additional file 1: Table S2). Three of these mutations were located within genes known to be active in the brain (MeCP2, EGFR and CEP112) while the other was intergenic and was not associated with a known regulatory element, such as a promoter region or annotated enhancer (Additional file 1: Table S2). The putative tumour-specific L1 mutations in MeCP2 and EGFR were validated via PCR (see below). The remaining two events identified by RC-seq could not be confirmed by PCR and hence their structures could not be fully elucidated. RC-seq read information however indicated that the putative L1 insertion in CEP112 involved an L1-Ta donor sequence, a long (102 nt) poly-A tail and a degenerate L1 EN recognition motif, suggesting potential TPRT-mediated L1 mobilisation, albeit without corroboration via PCR. The remaining putative L1 mutation was annotated as an older L1PA2 element, which are usually not capable of autonomous retrotransposition in humans [62], and also lacked an L1 EN motif. These features are consistent with a DNA rearrangement rather than a retrotransposition event. No somatic L1 sequence variants were detected in lower grade glioma samples.

### Structure and impact of a *de novo* L1-associated DNA rearrangement within MeCP2

In patient #2, a female, we identified a putative intronic L1 mutation in the methyl CpG binding protein 2 (MeCP2) gene by RC-seq that was confirmed by PCR (Fig. 1a-c, Additional file 2: Figure S1B-C, Additional file 1: Table S2). MeCP2 is an X-chromosome linked transcription factor necessary for neural differentiation and is defective in the neurodevelopmental disorder Rett syndrome [63]. MeCP2 binds and generally represses methylated DNA genome-wide, including the CpG-island present in the canonical L1 5′ promoter [64]. Sequence characterisation revealed that the L1 sequence belonged to the L1PA2 subfamily, was 5′ truncated and carried a 49 nt 3′ flanking region from its L1 donor element on chromosome 9. The L1 mutation site lacked an L1 EN recognition motif and TSDs, and incorporated a 58 nt genomic deletion (Fig. 1b, Additional file 2: Figure S1B), features strongly inconsistent with retrotransposition through TPRT. Further analysis revealed that the 3′ flanking region carried with the L1 comprised an *Alu* retrotransposon and that integration occurred into another *Alu* element. These features led us to conclude that this L1-associated event was probably driven by recombination of the *Alu* adjacent to the L1PA2 donor sequence with the *Alu* present in MeCP2.

Quantitative RT-PCR revealed significant (*p* < 0.008, *t*-test) reductions in tumoural expression of both of the two main MeCP2 transcript isoforms (Fig. 1a, d). Using additional primers specific to the L1 mutation, we performed qRT-PCR to evaluate whether the L1 generated a chimeric transcript with upstream or downstream MeCP2 exons. However, chimeric L1-MeCP2 RNA species were not detected by this assay (data not shown). WGS applied to patient #2 tumour and adjacent brain revealed tumour-specific copy number gain at the MeCP2 locus and an absence of single nucleotide variants or DNA rearrangements, other than the L1 mutation. Quantitative PCR measuring copy number of the genomic region deleted 3′ of the L1 mutation confirmed that the L1-mutant MeCP2 allele was amplified in the tumour as, despite overall amplification of the MeCP2 locus as detected by WGS, we identified copy number loss of this deleted 3′ sequence in the tumour. MeCP2 is known to regulate L1 activity by binding the methylated CpG-island present in the canonical L1 5′ promoter [64]. Therefore, as an evidence of a reduction in MeCP2 activity, we detected significantly higher (*p* < 0.0001, *t*-test) L1 mRNA abundance in patient #2 tumour versus adjacent brain (Fig. 1e), as well as significant tumour-restricted hypomethylation of the canonical L1-Ta promoter (*p* < 0.0001, *t*-test) (Fig. 1f). Replicate PCR performed on seven spatially disparate tumour foci detected the L1 mutation in all locations (Additional file 2: Figure S1C). These data suggest that the MeCP2 L1 mutation underwent copy number gain, was present in clonally amplified cells, and may have impacted MeCP2 expression and function throughout the tumour mass. Given these results, we propose transcriptional disruption by the L1 [65] as a plausible cause for reduced MeCP2 expression, although we cannot rule out the involvement of another mechanism.

### Structure of a tumour-specific L1 mutation within EGFR

**Fig. 2:**
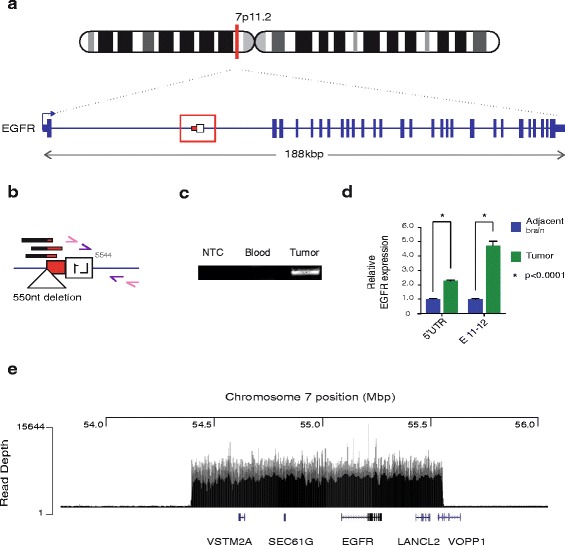
Characterisation of a somatic L1 mutation within EGFR. **a** Patient #8 EGFR mutant allele: a 0.5 kb L1-Ta sequence antisense to EGFR. Direction of transcription is indicated with *blue arrow*. **b** L1 mutation magnified view: RC-seq reads detected at the L1 3' terminus (*black/red bars*). The L1 mutation comprised a truncated fragment of L1 ORF2 (*white box*) and the 3′UTR without a poly-A tail (*red box*). A 550 nucleotide deletion at the integration site was also identified (triangle). Primers used for PCR validation are indicated as pink and purple arrows. **c** Mutation site PCR validation: Region comprising the EGFR-L1 5' junction was detected in patient #8 tumour sample. No amplification was detected when water (NTC) or genomic DNA from blood were used as template. **d** qRT-PCR measurement of EGFR transcription at its 5'UTR and exon 11-to-12 junction (E 11–12): The relative levels of RNA from both regions were significantly increased in tumour (*blue*) versus adjacent brain (*green*) samples. Data for each group were normalised to adjacent brain values, pooled and presented as mean +/− SEM (*p < 0.001, two tailed *t*-test, df = 10). **e** Amplified chromosome 7 region including EGFR: mapped read depth in EGFR region. Positions in Mbp are marked across the top horizontal axis. Read depth is reflected by the height of vertical lines as indicated on the vertical axis. Genes present in the amplified region are placed based on the locations of representative transcripts from UCSC Genes (hg19)

In patient #8, RC-seq detected a putative tumour-specific L1 mutation in the first intron of the epidermal growth factor receptor gene (EGFR) (Fig. 2a), a major oncogene amplified or otherwise altered in >60 % of GBM cases [6]. PCR validation and capillary sequencing showed a 5′ truncated L1 sequence of the L1-Ta subfamily and lacking a 3′ poly-A tail and TSDs, and here incorporating a 550 nt genomic deletion (Fig. 2b-c, Additional file 2: Figure S1D). Although these features are consistent with ENi L1 retrotransposition, we cannot fully exclude the possibility that this event arose via L1-associated DNA recombination. PCR upon multiple tumour foci suggested that the L1 mutation was clonally amplified (Additional file 2: Figure S1E). Unlike the MeCP2 L1 mutation, the EGFR L1 mutation did not appear to impact host gene expression. Indeed, EGFR expression was significantly up-regulated in patient #8 tumour versus adjacent brain tissue (*p* < 0.0001, *t*-test) (Fig. 2d). As EGFR structural and copy number variation is a common feature of GBM [6], we performed WGS on patient #8 tumour and blood samples, elucidating major (>50×) copy number amplification of EGFR and the surrounding genomic locus (Fig. 2e). Given the extreme copy number gain and the presence of discordant read pairs supporting a junction between the 5′ and 3′ ends of the amplified genomic segment, this event was likely to represent a double minute chromosome [66] containing EGFR. Further WGS data analysis suggested that the majority of additional EGFR alleles did not incorporate the L1 mutation, indicating that copy number amplification primarily drove EGFR induction.

### Whole genome analysis of patients #2 and #8

To place the PCR validated L1-associated mutations in MeCP2 and EGFR into a broader context of genomic abnormality, we analysed WGS data from patients #2 and #8 (Additional file 1: Table S5). For patient #2, we compared tumour tissue and adjacent tissue that appeared pathologically normal. Here we found that a significant portion of cells from the normal tissue did in fact contain tumour cells based on the presence of mutations at low variant allele fraction (VAF) in the normal tissue and at an increased VAF in the tumour tissue. For example, we identified a TP53 mutation (c.421C > T/p.Arg141Cys) present in 18.5 % of sequencing reads from adjacent tissue and 90.9 % of tumour reads. This mutation corresponded to rs121913343/COSM3719990 (dbSNP/COSMIC) but may have been somatic in this instance given the low VAF in normal tissue. Other potentially pathogenic point mutations in patient #2 included an established GBM mutation in the IDH1 gene at p.Arg132His [6]. We observe LOH over APC leading to at least two non-synonymous changes increasing in VAF to >95 % (rs139196838, rs459552). The tumour from patient #2 presented variable 5–10 fold amplification across 4q12 (Additional file 3: Figure S3A), which included the tyrosine kinase KIT, and tyrosine kinase receptors PDGFRA and KDR (VEGFR2) (Additional file 3: Figure S3B). Amplifications of this region occur frequently in GBM [67]. We also detected a focal ~2.7kbp deletion removing both copies of CDKN2A exon 2 inside of a larger single-copy region encompassing CDKN2A/B (Additional file 4: Figure S4). Finally, we detected copy number amplifications at the end of the q arm on chromosome X, which indicated one additional copy in two regions, one of which included MeCP2 (Additional file 5: Figure S5). This copy number increase provides a reasonable explanation for the 50 % decrease in MeCP2 expression in this tumour relative to the adjacent tissue, due to the aforementioned intronic L1 mutation in the amplified MeCP2 allele (Fig. 1).

As noted above, the tumour sample from patient #8 showed a remarkable amplification of EGFR (>50 fold, Fig. 2e, Additional file 6: Figure S6A), likely due to a double minute chromosome as observed in many other GBM cases [66]. Additionally this tumour contained a deletion surrounding CDKN2A/B, and an amplification of the RAS-related oncogene RAB14 on chr9q33.2 (Additional file 6: Figure S6B). There were few point mutations affecting known cancer or GBM-associated genes detected in this sample at appreciable (>10 %) VAF. The detected examples include a frameshift mutation of the histone methyltransferase and known tumour suppressor SETD2 (p.Asp14fs) that can activate TP53 and is necessary for DSB repair via homologous recombination [68, 69], and a putative splice donor site mutation affecting CIITA (class II MHC transactivator). Thus, the L1 mutation observed here in EGFR was likely a passenger to the main oncogenic transformation enabling tumorigenesis in patient #8 and occurred in an environment of impinged DNA repair.

### GBM cell lines rarely support L1 retrotransposition

**Fig. 3:**
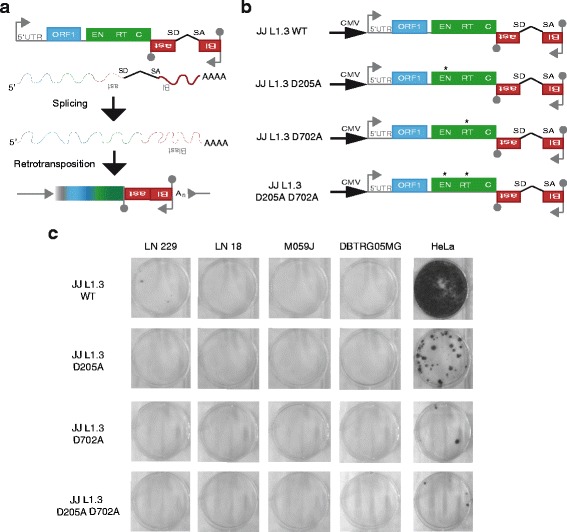
L1 retrotransposition rarely occurs in GBM cell lines. **a** Schematic representing L1 retrotransposition assay. A full-length L1 (L1.3) [61] is located upstream of the antisense oriented blasticidin resistance gene (*red boxes*). The L1 internal promoter is represented by an arrow on the 5'UTR region. Two L1 open reading frames (ORF1 and ORF2) are indicated by *blue* and *green boxes*, respectively. Functional domains of ORF2, endonuclease (EN), reverse transcriptase (RT) and cysteine rich domain (C) are also indicated. The blasticidin resistance gene is interrupted by an intron in the same orientation as the L1. Splice donor (SD) and splice acceptor (SA) sites are indicated. Polyadenylation signals are denoted by grey lollipops. **b** Schematic representation of retrotransposition assay constructs. JJ L1.3 WT contains an external promoter (cytomegalovirus promoter, CMV) upstream of a full length retrotransposition-competent L1.3 element [61]. *Asterisk* indicates missense mutation to abolish endonuclease activity (JJ L1.3 D205A), reverse-transcriptase activity (JJ L1.3 D702A) or both (JJ L1.3 D205A D702A). **c** Results of cell culture-based L1 retrotransposition assay. Each stained colony represents a cell where a retrotransposition event took place allowing the expression of the blasticidin resistance gene

To assess whether GBM cell lines support canonical or ENi L1 retrotransposition in vitro we performed an established cultured cell retrotransposition assay [43] on HeLa cells and four GBM cell lines (DBTRG-05MG, M059J, LN 18 and LN 229). This assay relies on the expression of a blasticidin resistance gene carried by an L1 reporter construct, where blasticidin is only expressed and confers resistance after L1 retrotransposition (Fig. 3a). Each cell line was transfected in triplicate with a set of 4 plasmids bearing different L1 sequences upstream of the antisense orientated blasticidin-resistance gene [60] (Fig. 3b); a wild type full-length L1 (JJ L1.3 WT) [61], an L1 with an EN domain missense mutation that abolishes L1 ORF2p EN activity (JJ L1.3 D205A) [23], an RT mutated L1 with no reverse-transcriptase activity (JJ L1.3 D702A) [59] and a double mutant L1 bearing both EN and reverse-transcriptase mutations (JJ L1.3 D205A D702A). No L1 retrotransposition events were detected for DBTRG-05MG, M059J or LN18 cell lines and very few events (<4 events per well) were detected for the L1.3 WT construct in LN 229 cells (Fig. 3c). Mobilisation of the RT mutant and double mutant L1 reporter was not observed in any of the GBM cell lines. By contrast, the positive control HeLa cells supported the expected “hot” L1.3 WT activity [43, 70, 71] as well as mobilisation of each L1.3 mutant to lesser extents. These data indicate that GBM cell lines typically only support very low or negligible L1 retrotransposition, in line with our RC-seq data obtained from patient tumour samples.

## Discussion

These experiments reveal rare L1-associated mutations caused by recombination or L1 ENi retrotransposition in GBM tumours, accompanied by an absence of TPRT-driven L1 insertions. Endonuclease-independent L1 insertions have been reported by several prior studies employing engineered L1 reporter constructs in cultured cancer cells or cells otherwise deficient for DNA damage repair factors [21–23, 72, 73], or through bioinformatic analysis of the human reference genome [24]. Unusual L1 integration sites identified by these studies incorporated, amongst other features, genomic deletions, deletions of the L1 3′ end and poly-A tail, absence of TSDs and absence of an L1 EN recognition motif. Here, by fully resolving the structures of GBM tumour-specific L1 mutations through RC-seq and capillary sequencing, we confirmed they lacked a recognisable L1 poly-A tail or TSDs, and incorporated genomic deletions. In the case of the EGFR L1 mutation, these features are suggestive of L1 ENi mobilisation, as primarily reported by others using engineered L1 systems in vitro [21–23, 72, 73] or, potentially, DNA recombination. Genomic abnormality at L1 mutation sites may also explain failure to PCR validate 2/4 observed putative tumour-specific L1 mutations. Notably, our WGS analyses elucidated mutations in key DSB repair factors, such as SETD2 in patient #8, as well as TP53 deficiency in patient #2. Thus, rare tumour-specific L1-associated mutations occur in GBM in a milieu of deficient DSB repair previously encountered for similar events in vitro [21, 23].

Although identified at first by RC-seq as a potential L1 mobilisation event, further characterisation of the MeCP2 L1 mutation indicated a probable DNA rearrangement event mediated by *Alu*:*Alu* recombination. Notably, the *Alu*-flanked L1PA2 donor sequence, located on chromosome 9, was 5′ truncated and did not encode a viable L1 ORF2p. The involvement of an older L1 family in a tumour-specific DNA rearrangement event is reminiscent of one of the earliest L1 mutations detected in cancer, in that case affecting the myc locus in breast tumour [74]. A lesser possibility is that the L1PA2 transduced its flanking 3′ *Alu* during ENi L1 mobilisation in *trans*, in agreement with a previous observation where 71 % of detected ENi L1 mobilisation events involved non-L1 DNA [24], and involved single strand annealing [72, 73, 75]. The tumour-specific L1 mutation in MeCP2 appeared to reduce the expression of this gene concomitant with copy number gain for the L1 mutant MeCP2 allele and, as suggested by increased L1 expression and reduced L1 5′UTR methylation, may have reduced MeCP2 function throughout the tumour mass, causing a molecular phenotype.

Notably, the EGFR L1 mutation provides an interesting example because, despite not having a direct effect on gene activity, it is one of very few tumour-specific L1 mutations noted in a major oncogene or tumour suppressor since the first such example was discovered more than 20 years ago by Miki et al. [9].

Previous studies employing high-throughput sequencing reported no tumour-specific L1 insertions in brain tumours [30–32, 35]. Iskow et al. did observe putative tumour-specific L1 insertions, though subsequent PCR validation experiments were unsuccessful [30]. Thus, although the atypical L1-associated mutations reported here represent the first PCR validated variants of this type in in GBM, our in vivo and in vitro results agree with prior reports of a lack of TPRT-driven L1 insertions in brain cancers (Table 1). At the same time, the absence of *de novo* L1 insertions in this context is intriguing, given frequent somatic L1 mobilisation via TPRT in neural cells [39–42], including glia [42]. To speculate, one explanation for consistently limited L1 activity in GBM could be that the relevant tumour initiating cells only support L1 retrotransposition in the context of a deficient DSB repair, perhaps due to the presence of L1 inhibiting host factors, including those affecting subcellular localisation of the L1 RNP [76]. This conclusion would disagree with GBM typically arising from de-differentiated neural cells. Still, given the high RC-seq coverage employed here, and evidence obtained using the L1 reporter system in vitro, we consider this explanation more appealing than tumoural heterogeneity obscuring TPRT-driven L1 mobilisation.

**Table 1:** Published analyses of L1-associated mutations in brain tumours

Study	Brain tumour types (sample count)	Sequencing method	L1 coverage (tumour)	Potential somatic L1-associated mutations (PCR validated)	Ref.
Iskow et al.	GBM (5), medulloblastoma (5)	L1-seq	3.6× - 6.4×	74 (0)	[30]
Lee et al.	GBM (16)	WGS	39.2×	16 (0)	[32]
Brennan et al.	GBM (42)	WGS	35.3×	0 (0)	[6]
Helman et al.	GBM (20)	WGS	40.8×	0 (0)	[35]
Tubio et al.	Glioma cell line (1)	WGS	42.6×	1 (0)	[31]
Carreira et al.	GBM (9), glioma (5)	RC-seq	43.2×–231.0×	4 (2)	–

## Conclusions

Although we conclusively find that L1 mobilisation is a rare event in GBM, our discovery of atypical L1-associated mutations in MeCP2 and EGFR demonstrates that L1 can otherwise contribute to GBM genome abnormality in key loci regulating neural cell differentiation and proliferation. Future experiments are required to ascertain whether this phenomena correlates with patient prognosis and whether potential DNA damage caused by chemotherapy or radiotherapy [77] activates L1 in recurrent GBM.

## Additional files

Additional file 1: Table S1. RC-seq output summary. **Table S2**. Polymorphic and somatic L1 mutations called by TEBreak. **Table S3**. List of somatic L1 mutations and PCR validation information. **Table S4**. GBM cell line transfection efficiencies. **Table S5**. Summary of point mutations and CNVs detected from WGS (XLSX 335 kb)
Additional file 2: Figure S1. Structure and detection of L1 mutations in MeCP2 and EGFR. (a) RC-seq versions: Capture probes from version 2 (V2, marked in blue) correspond to two sets of DNA oligonucleotides mapping within the 5'UTR and the 3'UTR of the L1 sequence (UTRs represented as red boxes). Version 3 (V3, marked in green) includes a single LNA probe for each UTR. The 5' end probes capture only the 5' junction of full or nearly full length insertions. The 3' end probes capture both 3' junctions of insertions of all lengths and 5' junctions of heavily truncated insertions. (b) Pre- and post- mutation site sequences for MeCP2: Pre-integration panel shows the sequence at the L1 mutation site as obtained from the human reference genome (GRCh37/hg19). A 58 nt deletion was detected after the L1 mutation (marked in green within box). Post-integration panel shows the 5' and 3' termini sequences of the L1 mutation and flanking genomic region. L1PA2 sequence is marked in red. The 49 additional nucleotides from an *Alu* are marked in grey. Micro-homology with the pre-integration genomic region is denoted (underlined). Slashes represent L1PA2 sequence not shown. Lowercase indicate mutations versus reference sequences. (c) MeCP2 L1 mutation PCR validation: DNA extracted from different regions of the tumour (denoted from A to G) was used as template for the amplification of MeCP2 5'end L1 junction. No amplification was detected when water was used as template (NTC). (d) Pre- and post- mutation site sequences for EGFR: Pre-integration panel shows the sequence at the L1 insertion site as obtained from the human reference genome (GRCh37/hg19). A 550 nt deletion was detected after the L1 mutation (marked in green within box). Post-integration panel shows the 5' and 3' termini sequences of the L1 mutation and flanking genomic region. L1-Ta sequence is marked in red. Slashes represent genomic or L1-Ta sequence not shown. Lowercase indicate mutations relative to reference sequences (L1-Ta, human reference genome). Untemplated nucleotides are represented in grey. (e) EGFR mutation site PCR validation: DNA extracted from different regions of the tumour (denoted from A to G) was used as template for the amplification of the EGFR 5' end L1 junction. No amplification was detected when water was used as template (NTC) (EPS 2005 kb)
Additional file 3: Figure S3. Copy number aberrations in the tumour sample of patient #2 on chr4q12. (a) Detailed view of the 4q12 region with aberrant and highly variable copy number changes in oncogenes PDGFRA, KIT, and KDR (VEGFR2). (b) altered region in the context of chromosome 4. Y-axis of both panels indicates the log10 fold-change in counts per million (CPM), X-axis indicates position on the indicated chromosome. Shaded regions indicate gaps in the chromosome reference sequence. (PDF 133 kb)
Additional file 4: Figure S4. Copy number aberration of CDKN2A on chromosome 9 in patient 2. (a) Detailed view of the CDKN2A region with the regional single-copy deletion and focal deletion of both copies indicated. (b) Altered region in the context of chromosome 9. Axes and shading are as described for Additional file 3: Figure S3. (PDF 130 kb)
Additional file 5: Figure S5. Copy number aberration of MECP2 on chromosome X in patient 2. (a) Detailed view of the MECP2 region with the regional single-copy amplification indicated. (b) Altered region in the context of chromosome X. Axes and shading are as described for Additional file 3: Figure S3. (PDF 135 kb)
Additional file 6: Figure S6. Copy number aberration of EGFR, CDKN2A, and RAB14 in patient 8. (a) Amplification of EGFR on chromosome 7 (see also Fig. 2e). (b) Amplifications on chromosome 9 including CDKN2A and RAB14. (PDF 151 kb)
Additional file 7: Figure S2. Retrotransposition assay flow cytometry gating parameters. Flow cytometry gating strategy for LN 229 cell line is shown as an example. Cells were distinguished from debris based on internal complexity (side scatter light area, SSC-A) and size (forward scatter light area, FSC-A). To identify single cells only, the original population was gated based on the size (FSC-A) and height (forward scatter light height, FSC-H) of the cells. Propidium iodide staining was used to distinguish dead and alive cells. GFP positive events were gated based on the absence of GFP signal from the untransfected live cell population. Flow cytometry analysis settings were established for untransfected cells and applied to transfected cells. (EPS 2683 kb)

## Abbreviations

APC: adenomatous polyposis coli gene
CDKN 2a/2b: Cyclin-Dependent Kinase Inhibitor 2a/2b genes
CEP112: Centrosomal protein 112 gene
CIITA: Class II, Major Histocompatibility Complex, Transactivator gene
DSBs: Double strand breaks
EGFR: Epidermal growth factor receptor
EN: Endonuclease
ENi: Endonuclease independent
GBM: Glioblastoma multiforme
IDH1: Isocitrate Dehydrogenase 1 gene
KDR: Kinase Insert Domain Receptor
L1 or LINE1: Long interspersed nuclear element 1
LM-PCR: Ligation-mediated PCR
LOH: Loss of heterozigosity
MeCP2: Methyl CpG binding protein 2 gene
ORF: Open reading frame
PDGFRA: Platelet-derived growth factor receptor alpha gene
RC-seq: Retrotransposon capture sequencing
RNP: Ribonucleoprotein particle
RT: Reverse transcriptase
TP53: Tumour protein p53 gene
TPRT: Target-primed reverse transcription
TSDs: Target site duplications
UTR: Untranslated region
VAF: Variant allele fraction
