# Supplementary figures and images for: Evidence for L1-associated DNA rearrangements and negligible L1 retrotransposition in glioblastoma multiforme
Source: Mob DNA. 2016 Nov 11;7:21. doi: 10.1186/s13100-016-0076-6 (PMC5105311; doi:10.1186/s13100-016-0076-6)

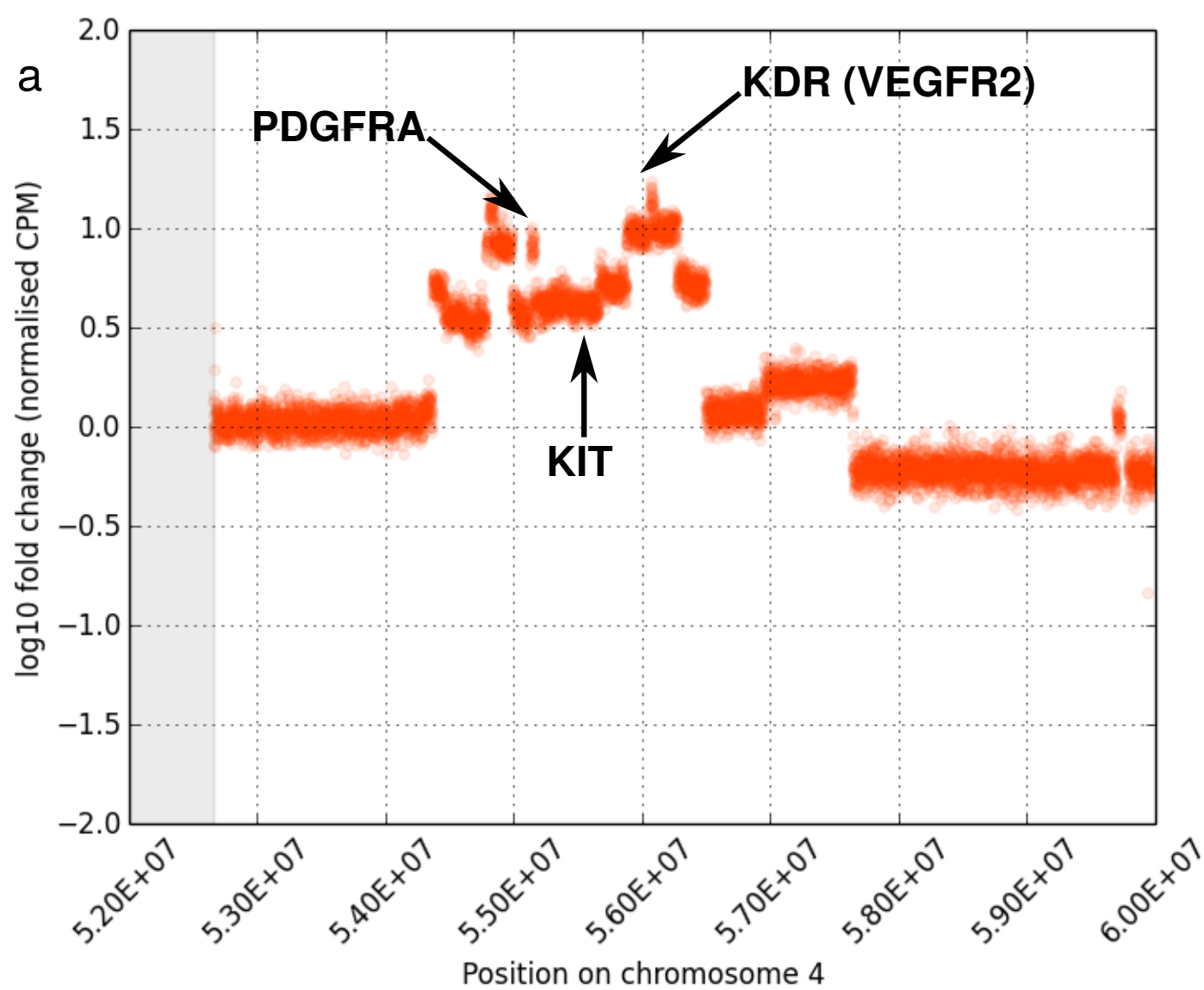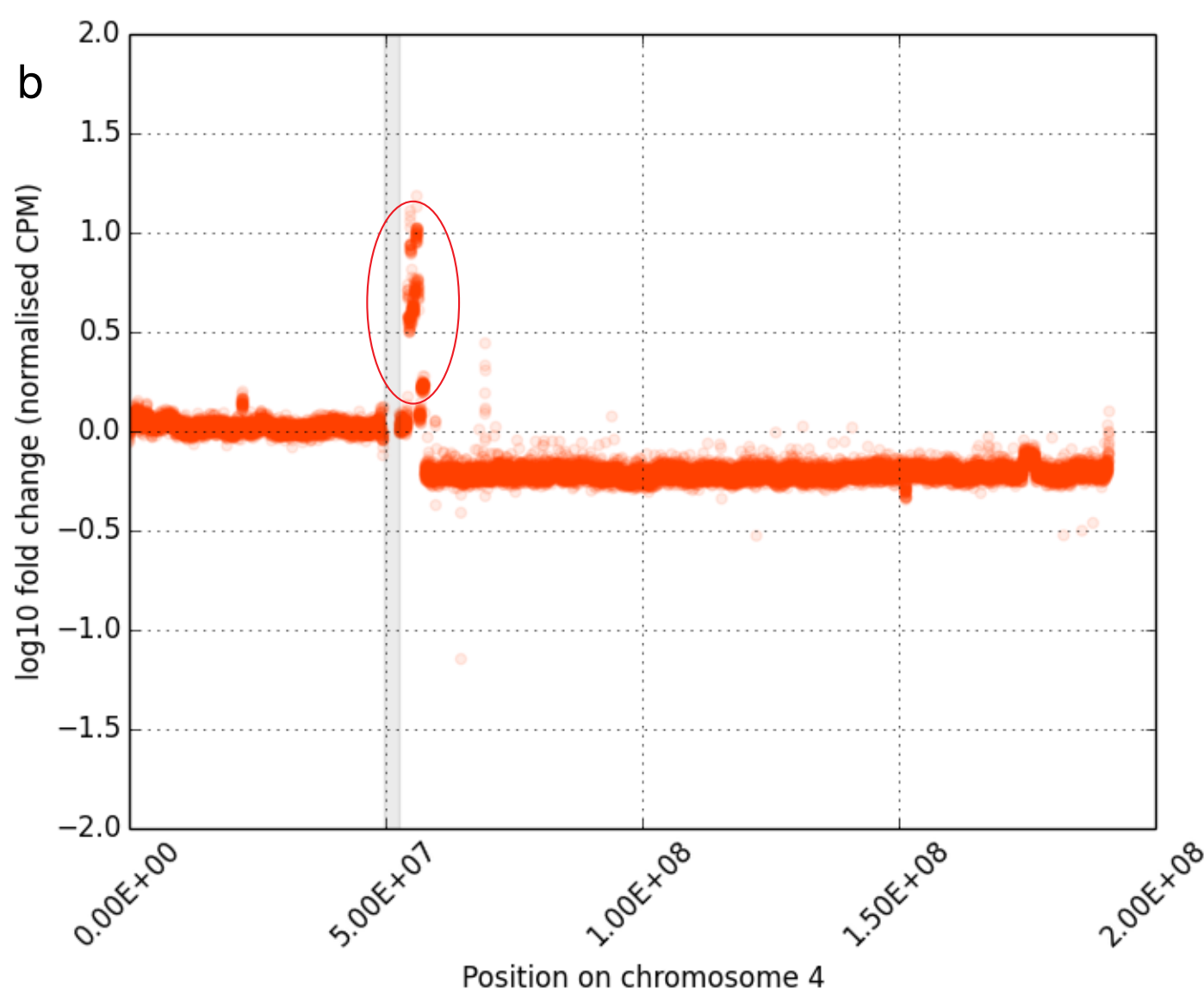

Supplement: Additional file 3: Figure S3. — Copy number aberrations in the tumour sample of patient #2 on chr4q12. (a) Detailed view of the 4q12 region with aberrant and highly variable copy number changes in oncogenes PDGFRA, KIT, and KDR (VEGFR2). (b) altered region in the context of chromosome 4. Y-axis of both panels indicates the log10 fold-change in counts per million (CPM), X-axis indicates position on the indicated chromosome. Shaded regions indicate gaps in the chromosome reference sequence. (PDF 133 kb) [file 13100_2016_76_MOESM3_ESM.pdf]

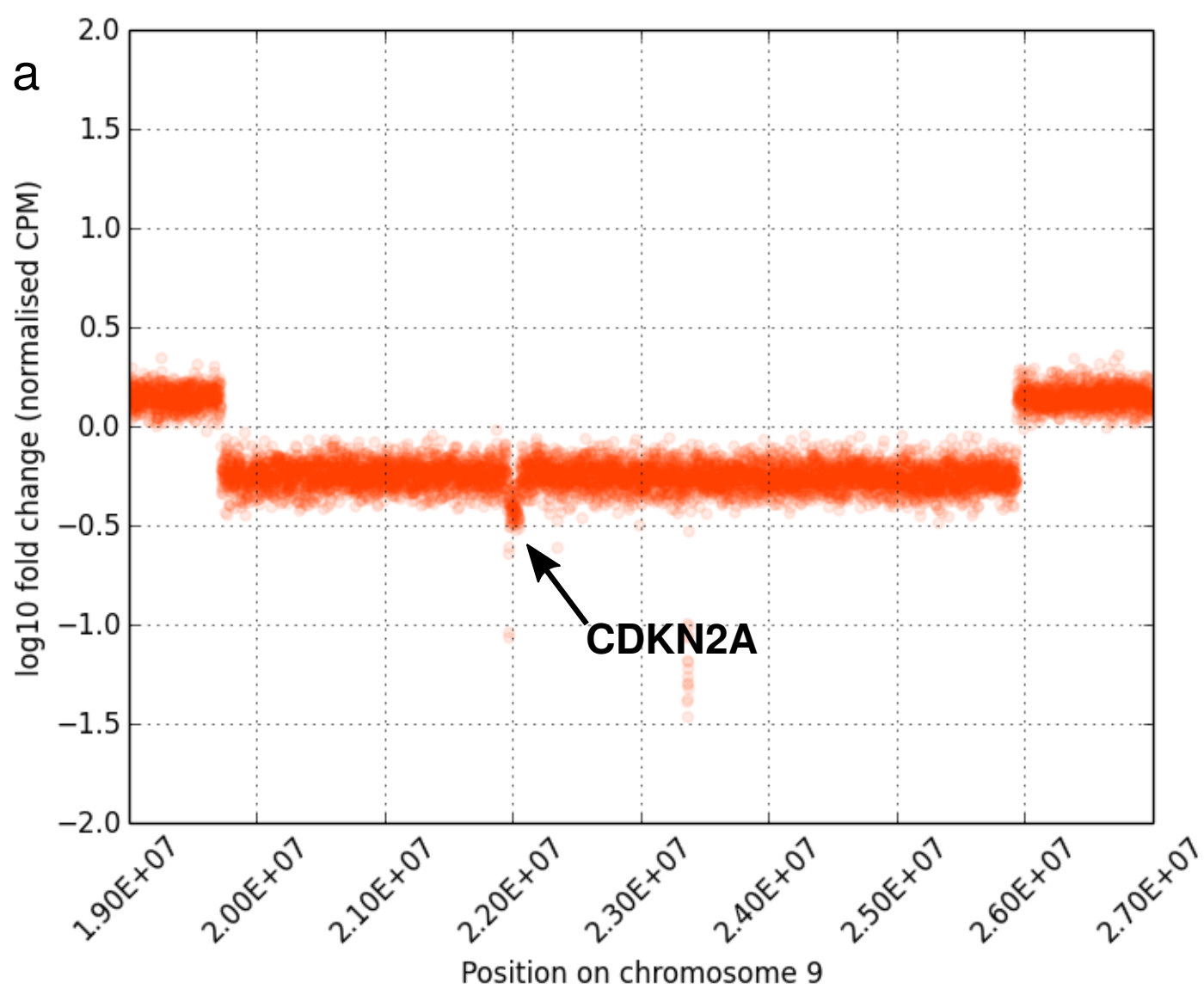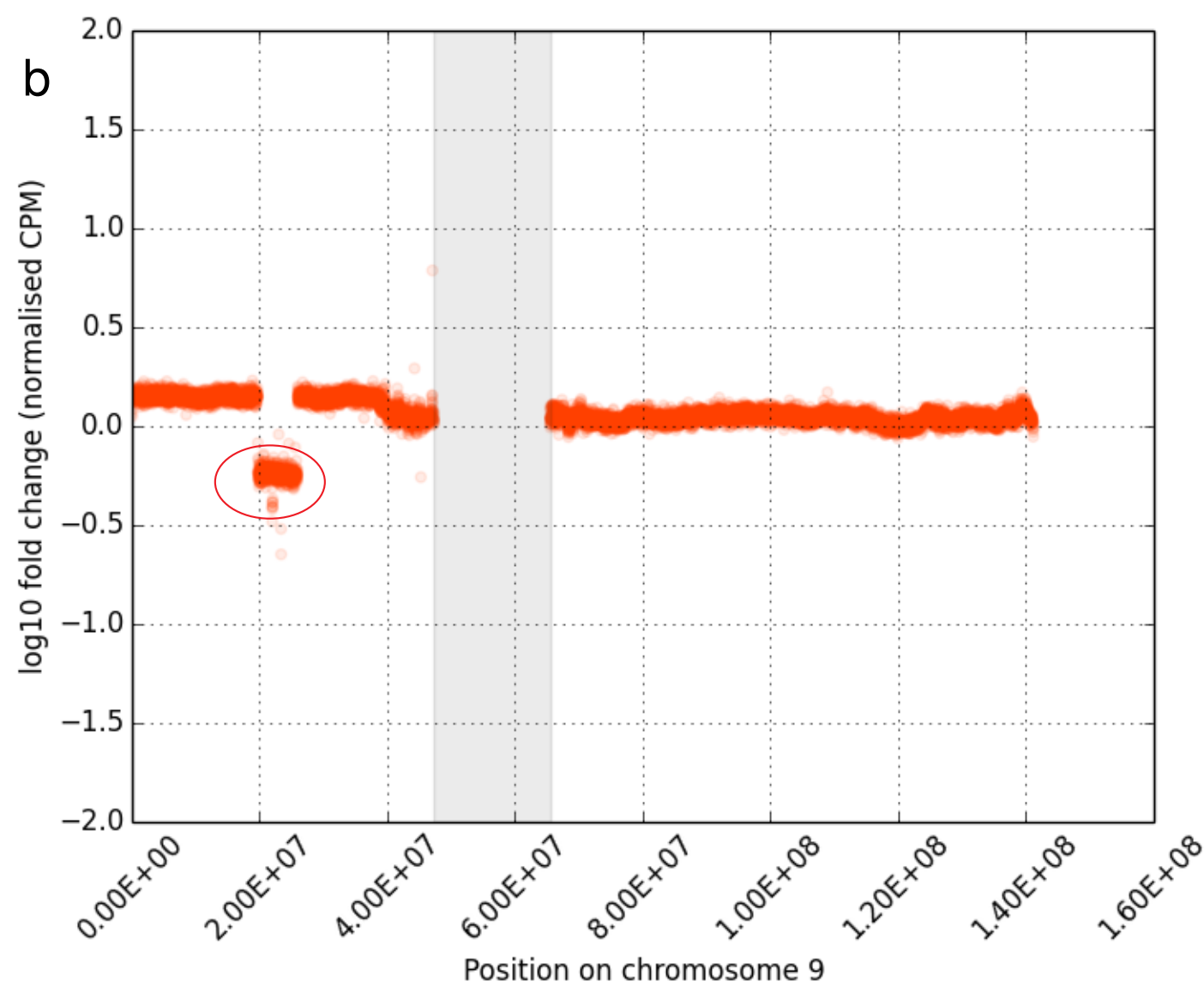

Supplement: Additional file 4: Figure S4. — Copy number aberration of CDKN2A on chromosome 9 in patient 2. (a) Detailed view of the CDKN2A region with the regional single-copy deletion and focal deletion of both copies indicated. (b) Altered region in the context of chromosome 9. Axes and shading are as described for Additional file 3: Figure S3. (PDF 130 kb) [file 13100_2016_76_MOESM4_ESM.pdf]

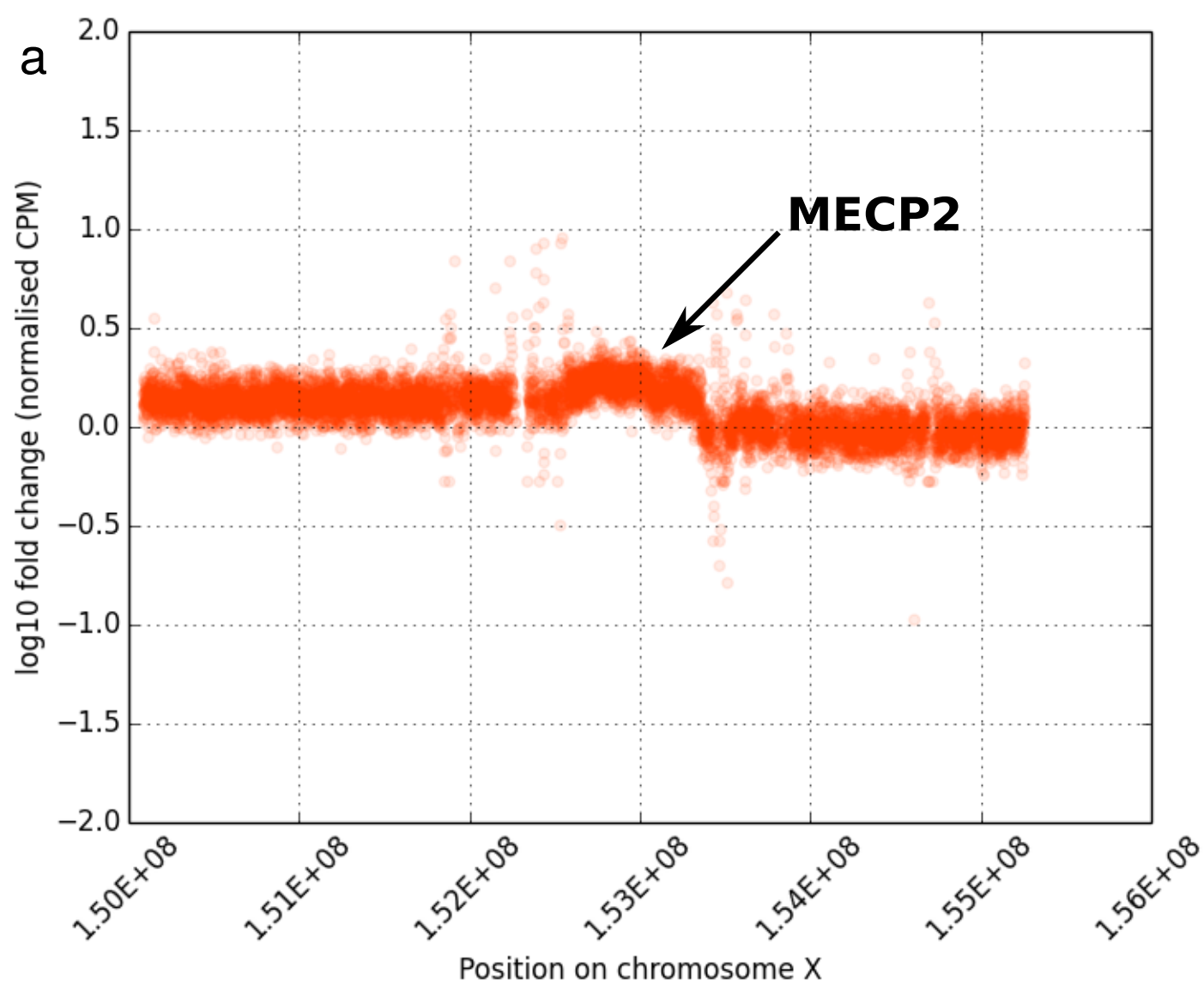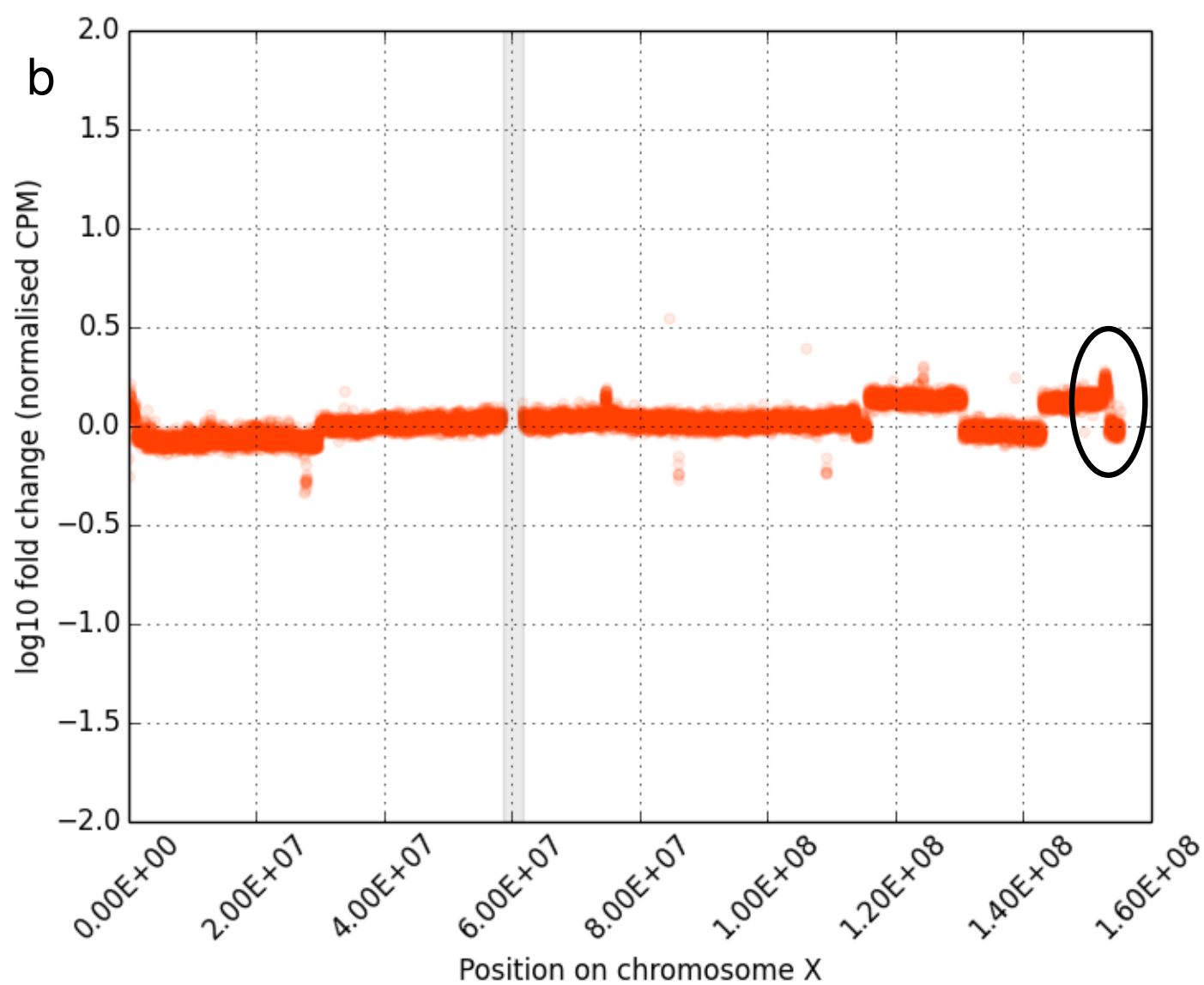

Supplement: Additional file 5: Figure S5. — Copy number aberration of MECP2 on chromosome X in patient 2. (a) Detailed view of the MECP2 region with the regional single-copy amplification indicated. (b) Altered region in the context of chromosome X. Axes and shading are as described for Additional file 3: Figure S3. (PDF 135 kb) [file 13100_2016_76_MOESM5_ESM.pdf]

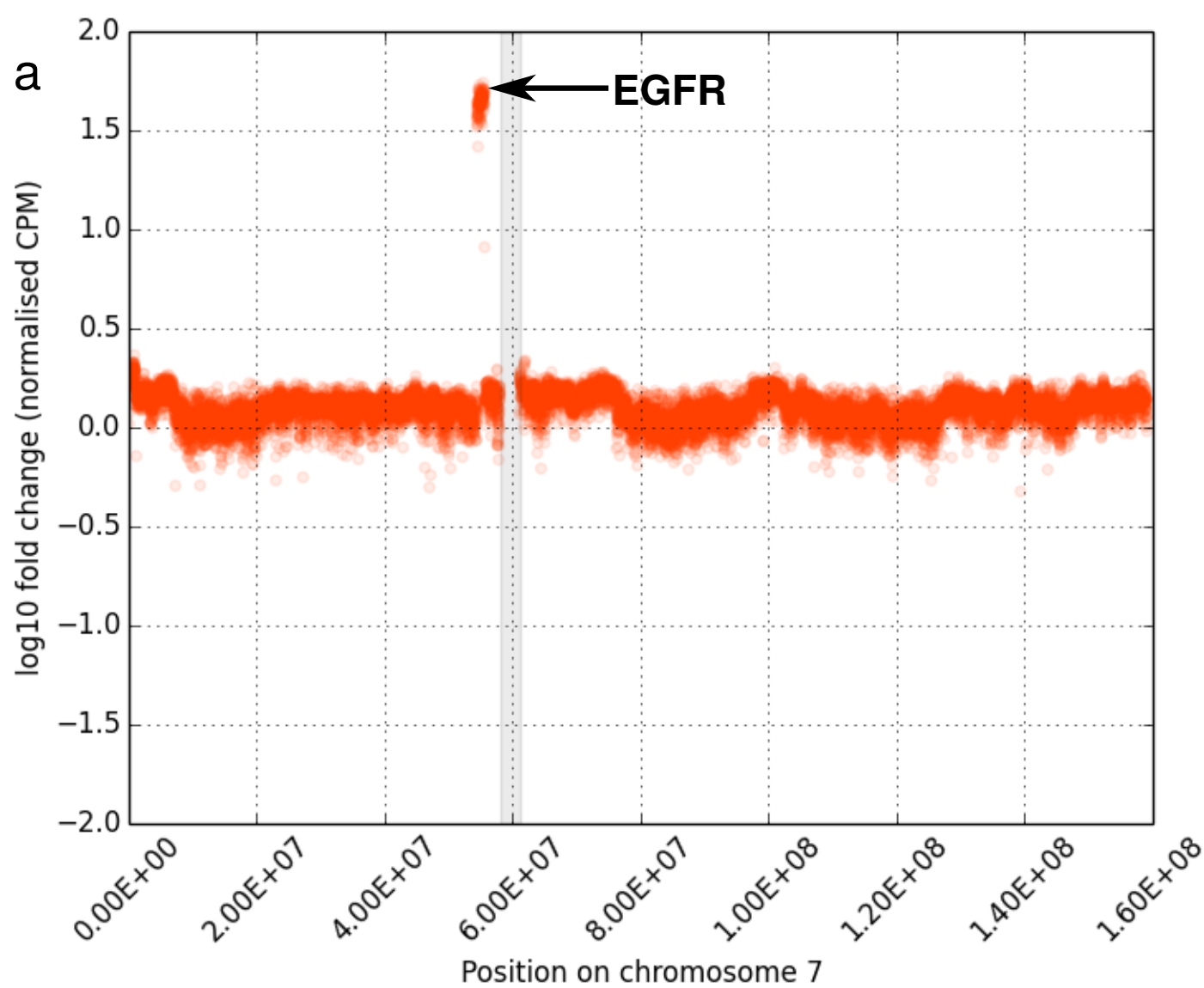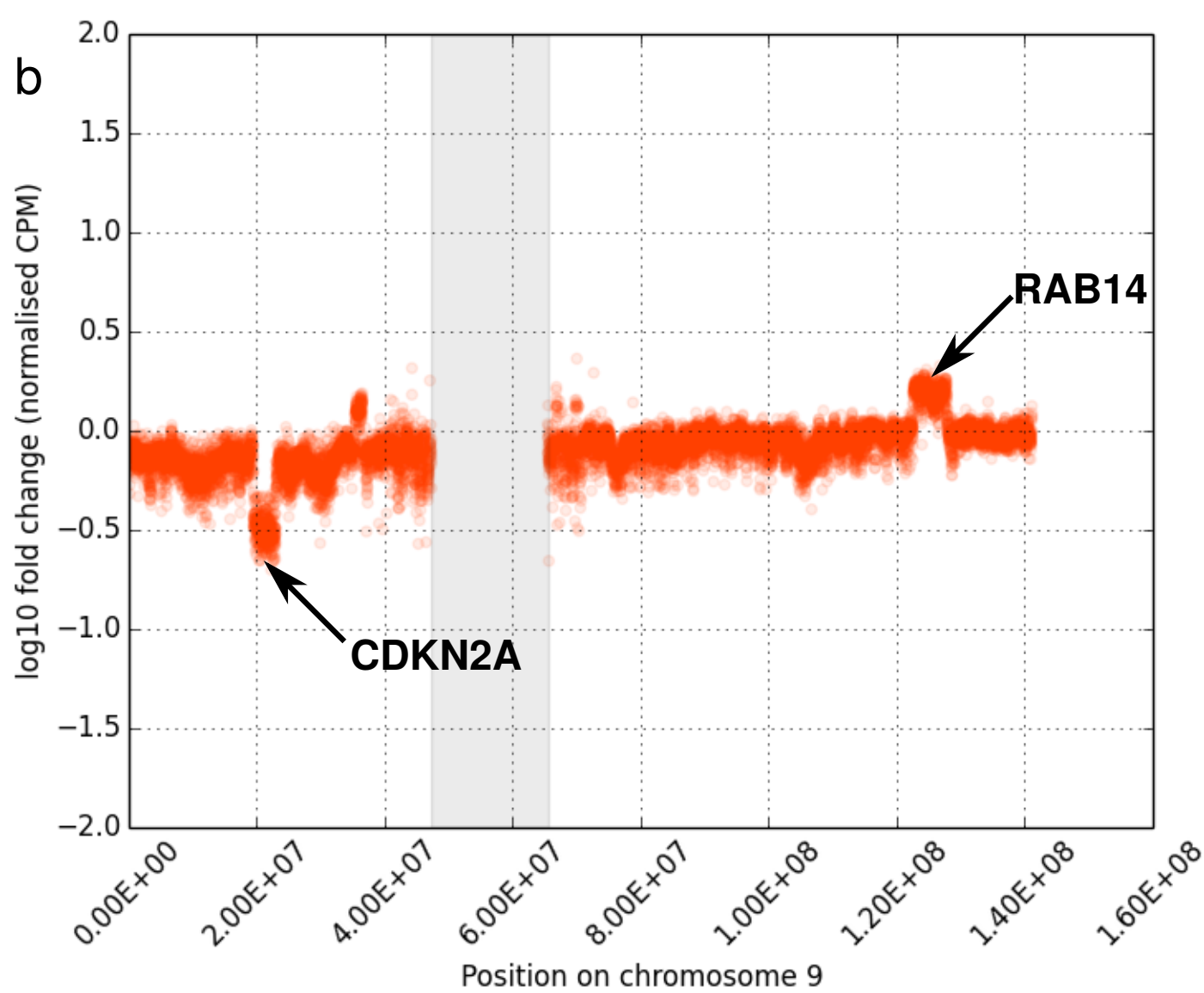

Supplement: Additional file 6: Figure S6. — Copy number aberration of EGFR, CDKN2A, and RAB14 in patient 8. (a) Amplification of EGFR on chromosome 7 (see also Fig. 2e). (b) Amplifications on chromosome 9 including CDKN2A and RAB14. (PDF 151 kb) [file 13100_2016_76_MOESM6_ESM.pdf]
